# SUPERRESOLUTION IMAGING OF CHROMATIN IN HEALTH AND DISEASE

**DOI:** 10.1093/geroni/igac059.105

**Published:** 2022-12-20

**Authors:** Melike Lakadamyali

**Affiliations:** University of Pennsylvania, Philadelphia, Pennsylvania, United States

## Abstract

Super-resolution microscopy has been playing an instrumental role in providing new insights into how the genome is folded and packaged inside intact nuclei in single cells. I will present our work on using super-resolution microscopy to visualize and quantify the spatial organization of chromatin with nanoscale spatial resolution in single cells. Our work has revealed that at the nucleosomal level chromatin is a disordered fiber composed of groups of nucleosomes packaged at varying densities, which we named nucleosome clutches. Despite the heterogeneity of nucleosome clutch organization, the size and packing density of nucleosome clutches is cell-type specific and correlates with cell fate. Our recent results also show that nucleosome clutches and chromatin nano-structure can be remodeled via chemo-mechanical cues. In particular degenerative chemo-mechanical cues during disease lead to aberrant chromatin nano-structure and loss of mechano-epigenetic memory, potentially leading to alterations in cell phenotype.

